# The experimental psychology behind coordination

**DOI:** 10.1007/s40656-026-00724-7

**Published:** 2026-03-18

**Authors:** Flavia Padovani

**Affiliations:** English & Philosophy Department, Drexel University, Philadelphia, PA 19104 USA

**Keywords:** Reichenbach, Coordination, Construct validity, Early experimental psychology, Measurement

## Abstract

In the philosophy of science, the *problem of coordination* concerns the difficulty of linking abstract theoretical concepts with their empirical counterparts—that is, determining how theoretical terms such as “mass” or “temperature” correspond to observable phenomena. A closely analogous issue arises in psychology in relation to the concept of *construct validity*, which addresses the extent to which an empirical measure accurately represents the theoretical construct it is intended to assess. From the perspective of contemporary literature on coordination and validity, Reichenbach’s interpretation of coordination stands out as uniquely engaging, not only because it offers an account involving issues of particular significance within the philosophy of measurement, but also because it originated in a less limited conceptual landscape than it is generally assumed. In this paper, I will emphasize some historical aspects underlying Reichenbach’s formulation, particularly his early engagement with experimental psychology, which underpins and strengthens the potential of the connection between the concept of coordination and that of construct validity.

## Introduction

Generally speaking, the idea of “coordination” involves the process or act of connecting theoretical concepts or terms with phenomena and it has typically been used to refer to the problem of applying abstract scientific theories to the physical world by specifying how mathematical equations can be linked with empirical observations. Many recent discussions of this idea in the philosophy of science owe much to the debate carried out by figures such as Ernst Cassirer, Moritz Schlick, and Hans Reichenbach around the beginning of the past century, but it is Reichenbach’s account that is generally associated with this idea today. To be sure, Reichenbach’s early approach to the problem of coordination reflects a broader epistemic concern: how to account for the notion of empirical validity. For him, in alignment with his initial neo-Kantian project, the kind of “certainty” we need cannot proceed from experience alone, nor can we merely rely on an unjustified assumption of the validity of physical laws, for these contain parameters that are not already given and without which theories would not be linked to the empirical sphere. Thus, from the perspective of contemporary literature on this topic, Reichenbach’s interpretation stands out as uniquely engaging, as it offers an account of coordination involving issues of particular significance especially within the philosophy of measurement.^1^ Additionally, as a philosophical figure, Reichenbach appears well positioned to carry out investigations bridging traditionally distinct strands of research. The peculiarity of his approach is that it is rooted in analyses of empirical theories, specifically innovations in physics, which is apparent in his way of addressing inferences and theory testing in his early work. Although not always thoroughly developed, many epistemological advances in his first writings were in part overshadowed by the success of his subsequent most influential work, *The Philosophy of Space and Time* (1928). These advances are often broadly related to, if not inspired by, his approach to scientific practice and—especially initially—his interest in wireless telegraphy and radio technology.

As a matter of fact, recent literature in psychology reveals a significant affinity between the notion of “coordination” and that of “construct validity”, a fundamental concept in psychological measurement, which addresses the extent to which a test or tool accurately measures the theoretical concept it is intended to measure. In both contexts, broadly speaking, the question amounts to the following: how do we ensure that what we measure truly represents the theoretical construct under study? In both cases, the coordination between the construct and its indicators is not self-evident, and it relies on theoretical reasoning, empirical testing, and interpretive judgement. In some sense, given Reichenbach’s logical-empiricist conceptions and initial inclinations, the analogies between these two concepts seem natural, and even more so considering that Paul Meehl, one of the two authors of the seminal piece “Construct Validity in Psychological Tests” (1955), had an appointment to the Minnesota Center for Philosophy of Science at the time in which he was working on this piece, and was admittedly influenced by logical empiricism, as he noted in the Preface to his (1991). In this paper, I will consider these analogies by looking at the motives underlying Reichenbach’s formulation of this problem primarily by exploring some of his early work in less known domains.

Notably, his initial attempts to provide a solution to the problem of coordination reveal several interesting traits that have in part been overlooked in the literature. This was especially true during the initial phase of the revival of scholarship on his early work—often relying on selected archival material—when his original views on coordination were predominantly construed as being primarily inspired by Schlick’s and basically prompted by an effort to come to terms with the theory of relativity.^2^ While Schlick’s work certainly contributed new elements to Reichenbach’s later interpretation, his understanding of coordination was already well established, at the core, in his previous writings and reflections. Reichenbach only incorporated these new elements into an already quite developed view.

As it turns out, Reichenbach first elaborated on the notion of coordination in the framework of his doctoral dissertation, *The Concept of Probability in the Mathematical Representation of Reality* (1916), well before he had even read any work by Schlick. In those years, his interests were broadly oscillating between politics, philosophy, and the sciences. Furthermore, in his first semesters at university, Reichenbach was exposed to (and showed interest in) what he referred to as “scientific psychology” and conducted empirical research in psychology as part of his university coursework. During the First World War, he was involved in the creation of an aptitude test for telegraphists, and in the years immediately after the war, he worked on amplifying valves in the industrial sector of wireless telegraphy while attending Einstein’s lectures on statistical mechanics and relativity theory in Berlin. It is amidst this intellectually stimulating environment and with a practice-oriented mindset that Reichenbach drafted his Habilitation thesis, which was later published with the title *The Theory of Relativity and *A Priori* Knowledge *(1920). It is in this work that he further developed his idea of “cognition as coordination”, which has gained some prominence in present discussions in the philosophy of science—an idea that eventually culminated in an account of theory change (and, soon afterwards, of confirmation through theory testing).

In the next two sections, I will first describe little-known elements of Reichenbach’s formative years, particularly in relation to his early engagement with experimental psychology and in connection with how his notion of coordination came about (Sect. 2), and I will then highlight the peculiarity of his original (and often creative) approach (Sect. 3). As I will suggest in the concluding Sect. 4, these elements can offer fruitful insights supporting the analogy between the notion of coordination and that of construct validity in several salient respects. Specifically, Reichenbach proposes an interesting solution to the problem of coordination involving individuating, defining, and measuring physical magnitudes by progressively shaping the material obtained from perception using coordinating principles. His model requires constant refinement, i.e., an iterative procedure, systematic empirical confirmation, and a criterion specifying when a coordination is successful depending on the context of use, or when it is no longer corroborated by empirical evidence, which leads to a radical change in the coordination. These considerations reinforce the importance of the choice of coordination in theory testing and show how properly addressing problem of coordination can impact conclusions about empirical patterns drawn from different sets of hypotheses—which are, as I will suggest, features that have also been variously discussed in the secondary literature in relation to “construct validity”.

## The originality of Reichenbach’s background

Reichenbach’s most well-known primary research interests encompass probability and space–time theories, which are also the focus of his two theses: his doctoral dissertation on probability (1916) and his subsequent Habilitation thesis on relativity theory (1920). Throughout both works, the concept of coordination emerges as a key concept, and in both cases, it is motivated by the necessity of reconciling scientific advances with their philosophical underpinnings. Before analyzing this concept in his early work in Sect. 2.2, and its refinement including an account of theory testing in Sect. 3, it is worth considering some other of his early activities, as an aspect that does not seem to be coincidental in the way in which Reichenbach addresses problems related to measurement is that it overlaps with in his interest in experimental psychology in his first academic years, as well as in his work in the sector of wireless telegraphy and radio technology during and after the First World War (Sect. 2.1).

### Reichenbach’s early connection to experimental psychology and technological interests

Reichenbach embarked on his academic journey with a focus on engineering, but before long, he shifted his focus to philosophy and to problems related to psychology broadly construed. All these various interests were interwoven in Reichenbach’s initial work, although this has not been sufficiently highlighted in the secondary literature on in his work, up to date.

It is worth noting that although psychology progressively grew as a discipline within philosophy in the eighteenth and early nineteenth century, it was not institutionally separated from philosophy in German universities until the early 1930s.^3^ Furthermore, especially in the second half of the nineteenth century, psychology integrated insights from various disciplines, including physiology and physics. Situated at the crossroads of multiple academic fields, the emergence of psychology faced numerous challenges, particularly in defining its methodological and disciplinary identity, as it wavered between the empirical approaches of the natural sciences and the more traditional approaches of its “mother” discipline, philosophy. The creation of laboratories and institutes marked a turning point in Germany’s academic context, opening new avenues for psychological research that was carried out in more scientific fields (including medicine), the ones that received most fundings.^4^ This eventually led to the creation of psychological institutes and laboratories,^5^ which fueled the development of a more scientific, experimental strand of psychology: empirical research and practical experimentation became effective means to reject baseless conjectures from speculative philosophers.^6^

As is clear, the new psychological discipline that was emerging spanned a broader spectrum of topics than the one we are familiar with today. As Andreas Kamlah wrote (1987, p. 100), back then, “the investigation of the mind included, besides such subjects as emotions, perceptions, and volitions, concept formation and logical inference. Thus, probability, too, became a subject of psychology. While psychologists of our day use probabilistic methods as an instrument for research, for nineteenth-century psychologists it was a subject of direct investigation.” It is indeed this type of “direct investigation” that eventually became vital to Reichenbach’s academic pursuits. Prior to developing an interest in probability theory, specifically within the framework of the physical sciences, in the late summer of 1911, Reichenbach contemplated the idea of studying “scientific psychology”, as he wrote to a fellow member of the Free Student Movement, Kurt Lewin, a figure that would become not just important for Reichenbach, but also influential in the field of applied psychology (Padovani, 2013, 2022). When he enrolled at the University of Berlin, during the winter semester 1911–12, Reichenbach attended the lectures of one of the most prominent experimental psychologists of that time, Carl Stumpf.^7^ Stumpf wielded significant influence not only in shaping the landscape of experimental psychology at the beginning of the past century,^8^ but also in pioneering experiments with tones, as well as in the psychology of acoustic perception, which would soon turn out to be particularly interesting for Reichenbach.^9^ One year later, now enrolled at the University of Munich, Reichenbach engaged in completing empirical psychological coursework under Aloys Fischer, a former student of Theodor Lipps. Reichenbach’s project revolved around themes related to character formation, exploring psychological and philosophical aspects of human behavior and development.^10^ During his final university year, after failing to secure the doctoral supervision of the famous neo-Kantian philosopher Paul Natorp, Reichenbach attempted—also unsuccessfully—to write his doctoral thesis under Georg Elias Müller (Padovani, 2013, p. 99), a prominent experimental psychologist who was also the head of the psychological laboratory in Göttingen.^11^

Another notable chapter in Reichenbach’s activities during this decade revolves around his curiosity about radio technology and his varied contributions to the field of wireless telegraphy. During World War I, he served for nearly two years in the Signal Corps of the German army stationed in Neuruppin, not far from Berlin.^12^ There, he was employed with the specific task of developing an aptitude test for telegraphists, bringing his scientific and practical competencies to the front like many others young scientists and psychologists, who were also in charge of developing tests for specific military purposes (Lang & Corstjens, 2022). Mitchell Ash’s characterization of this tendency is worth quoting at length:

The First World War saw the emergence of the technological battlefield, and it also marked a turning point in the interaction of technology and basic science. In numerous ways scientists demonstrated the usefulness of basic research by employing laboratory instruments and techniques to solve military problems, from the development of sound-ranging devices in physics to that of poison gas in chemistry. Psychologists in Germany participated in this process by adapting psychophysical measurement to skills testing for the selection of communications specialists, pilots, and drivers. The focus of these efforts was on the ‘human factor’—on the human organism as a functioning part of a machine. Practitioners in the new field called ‘psychotechnics’ searched for the machine operators whose skills were best suited to the task in question—whose reactive idiosyncrasies, that is, interfered least with efficient functioning. (Ash, 1995, p. 188)

Remarkably, Reichenbach’s collaborators in this project were Kurt Lewin and Otto Lipmann, one of the first psychologists to apply the new research tendencies in psychology to industrial problems and co-founder, with William Stern, of the Institute for Applied Psychology in Berlin, as well as of the *Zeitschrift für angewandte Psychologie*.^13^ Reichenbach was responsible for overseeing the construction of a machine on which the test was supposed to run^14^ but he apparently often let his genuine theoretical and scientific interests prevail over the ultimate reason for that project.^15^

After the end of the war, Reichenbach was employed by the radio company Erich F. Huth, *Gesellschaft für Funkentelegraphie*, where his job was mostly and variously covering matters surrounding amplification, with tasks ranging from writing reports on other works on products similar to the ones manufactured by the company (such as checking the extent of patent infringements), to other more technical-theoretical writings on amplifying valves and on statistical procedures for their assessment.^16^ Among the wide array of reports, hand-written notes, and numerous writings on these topics in the Hans Reichenbach Papers Collection (HR),^17^ one is particularly interesting as it is indicative of Reichenbach’s attitude towards understanding different measurements of a magnitude. The document, a memo dated November 12, 1919 (HR 018-14-01), analyzes alternative ways of assessing the magnitude of a signal, depending on its power, voltage, or current, which is indispensable in several devices in order to enhance signals for better transmission and reception. Reichenbach begins the memo by listing three distinct definitions of the degree of amplification (*Verstärkung*) *W*: 1) the one he refers to as “Barkhausen”, according to which *W* is expressed as the ratio of the primary power in the input transformer to the output power in the telephone; 2) the one that he names the “Aviator Test Department” (that he presumably obtained from or used while serving in the Signal Corps), which expresses *W* as the relation between the power without amplifier with respect to the power with amplifier; and 3) the Schottky, for which *W* is provided by comparing the maximum power of the detector circuit with the output power in the telephone.

In evaluating these definitions, Reichenbach emphasizes how the second and the third are in principle not adequate because they “involve constants of the receiver”, which means that they “do not measure the amplifier alone, but rather the whole system: receiver-amplifier”. These definitions could be useful and indeed have some advantage, Reichenbach observes, “for military purposes, where the goal is to determine the most suitable amplifier for a particular receiver type”, but they are not effective in capturing the degree of amplification alone. The first definition could perhaps be a good candidate for that, but as it turns out, it is also “problematic”: it should first be clarified whether this measure is unambiguous (*eindeutig*). To remove any ambiguity, in fact, we should first assume that the amplification factor is strictly dependent on its argument (the ratio between the two powers), i.e., that it is a univocal (*eindeutig*) function of it. However, this is not guaranteed in the Barkhausen definition, as measurements from the aviator tests department have shown “that amplification decreases with absolute volume”, thus the amplification factor cannot be considered regardless of the power level.^18^

Reichenbach then examines further aspects of the input and output signals across three types of amplification, that is, depending on power, voltage, or current. Determining if any of these three types could consistently yield the same amplification factor given the same initial value is necessary to resolve the issue of ambiguity raised above and therefore obtain, as he writes, a “definition of the degree of amplification that is independent of factors outside the amplifier, because the phase in the input circuit is influenced by the detector resistance or, possibly, by the resistance of an audio tube.”

The final part of the memo discusses some sort of protocol involving three different engineering experiments that would provide three resulting amplification measurements. Although the memo is not conclusive in solving the issue at stake, it is quite revealing of Reichenbach’s frame of mind, and of how his engineering-experimental work cannot be separated from his philosophical reflections. In this particular case, it is remarkable how the analysis of the initial three definitions involves not only whether they actually provide a univocal definition of amplification but also addresses—albeit *en passant*—their effectiveness (and validity) in specific contexts. In other terms, it is not only their accuracy that matters, and whether what is obtained is in effect the sought-after magnitude, but also the idea that different approaches to those definitions could all be *equally* valid or justified, in their own ways, *in practice*.

### Errors, measurement, and the probability machine

Reichenbach’s doctoral thesis (1916) was essentially completed before the outbreak of Word War I and, consequently, before he was employed in the wireless telegraphy sector, and yet it reveals quite a similar attitude. This thesis focussed on the idea that it is possible to identify and define a concept of probability suitable for the mathematical representation of reality. His ultimate aim was to provide an account of probability that (to his mind) was necessary to complement the old Kantian system (Eberhardt, 2011).^19^ Concerns broadly related to quantification, e.g., the identification and measurement of quantities, were a constant trait of Reichenbach’s early work, as we have just seen. Setting aside the wider epistemological context in which he sought to frame his probability interpretation, the main problem for him was to provide an *objective* account of probability on which precise measurements could rely, and even be conceived (Padovani, 2021). At the same time, Reichenbach insisted that a comprehensive account of probability should express how the occurrence of an event is connected with a state of affairs in the *actual* world, rather than merely reflecting our subjective knowledge, as was common in more classic epistemic or subjective approaches to probability (Kamlah, 1987). In other words, probability statements should convey something certain about the world itself, not about our current state of knowledge of it.

As much as these issues could *prima facie* look disconnected from the type of activities that Reichenbach carried out before and after the war, reflections on probability were variously intertwined with debates at the core of psychological and psychophysical research at that time, as briefly noted earlier. Two key figures who authored the treaties on probability most extensively discussed in Reichenbach’s dissertation (i.e., Carl Stumpf and Johannes von Kries) were also physiologists and / or psychologists, as many other authors of fundamental contributions to the foundations of probability, like Alexius Meinong, Karl Marbe, August Fick, and Gustav Fechner, that Reichenbach either referred to in his thesis or wrote notes about while working on it. This is not surprising, considering that the history of psychology as a discipline, as Slaney recently put it, “is in many ways a story about psychological measurement, about attempts to represent psychological attributes quantitatively and then determine whether and the extent to which particular quantitative representations constitute “good” measurements of the psychological attribute in question” (Slaney, 2017, p. 29), for which an ability to deal with measurement error is clearly imperative.^20^

Reichenbach’s dissertation’s second chapter specifically examines the theory of errors as part of a broader effort to develop a unified account of probability theory including also another separate area, that of games of chance. Interestingly, the chapter begins with the construction of a device, inspired by a game of chance invented by von Kries (1886), the *Stoßspiel*, that Reichenbach calls “the probability machine”.^21^ The apparatus he imagined consists of a tape with very narrow and equal-sized black and white stripes, moving across two fixed rollers that are perpendicular to their direction of motion. In addition to the tape, a second component, a cylinder, is placed vertically above the tape and contains a sort of pointed projectile shooting in the direction of the tape. When the projectile is shot downward, it pierces the tape. His idea is that, by increasingly repeating the procedure a sufficiently large number of times while progressively decreasing the width of the stripes, we will observe a uniform distribution of the punctures over the two sectors of the tape. Reichenbach’s analysis of this mechanism, and of the conditions for objective probability claims that he extrapolates, are supposed to allow for the formulation of his so-called “probability function”.^22^ Now, we will not pursue his reasoning further, which rests on assumptions regarding the shooting process rather than the geometrical properties of the partition of the tape (differently than in Kries’s game)^23^ but once again we can observe his particular attitude towards elaborating theoretical concepts by reflecting on practical scenarios and concrete instances.

As we have seen in the case of his memo on the measurement of amplification, Reichenbach is well aware that *exact* measurement is in principle not possible. Apart from our perceptual limitations as humans, as there are no closed systems in nature, the (potentially) infinite influences that could have an impact on instruments should also be considered. Every measurement value, in some sense, results from a combination of influences that could be perturbing a functional or causal connection typically described by a physical equation.^24^ So, the problem is how and on what basis can we decide which is the “correct” value among the ones obtained. According to Reichenbach, we need a general law expressing the distribution of values in space and time, what he calls the “principle of lawful distribution”. This principle, which is supposed to complement the principle of causality, states that the empirical distribution of values has a convergence limit and thereby “warrants” that the observed convergence (which is obtained “with certainty”) essentially corresponds to the true one.^25^

Independently of the meaning of this principle and of whether his attempt at defining it in a satisfactory way was successful (it was not), what is significant here is Reichenbach’s motivation. Ultimately, it is the idea of reliability of measurement procedures, necessary to guarantee the validity of our scientific theorizing, that lies behind his early conceptualization of probability. His early reflection on coordination arises precisely in this context and it is triggered by the realization that coordinating abstract structures to reality is not as easy a business. Mathematical objects and judgements are fully and univocally specified but the same is not true for empirical judgements. The point he wants to make is that every empirical claim basically “*asserts the validity of a particular mathematical structure of reality*”. As he maintains (1916, p. 113; emphasis in the original):

“Validity” seems to me to be the word that best characterizes this situation. It would be incorrect to speak of a correspondence between the mathematical structure and reality; this can never be claimed, since real events are always governed by an infinity of equations. There are, as has been mentioned earlier, no closed processes in nature. Hence, the mathematical system will only ever approximately represent reality. But it has to be said that the influences that crucially determine the events are represented in the equations. […] If we are to have physical judgments, then an approximate representation of reality by mathematical equations has to be possible. In each such circumstance, we will speak of the validity of particular mathematical equations for reality. […] The possibility of physical knowledge has thereby been traced back to the assertibility of numerical approximations.

In other terms, if we were not able to deal with errors and understand how approximation works, we would not be able to guarantee a convergence “with certainty” and, thus, determine whether or not we have picked the correct measurement value, and finally whether our scientific theories are well grounded. Notice, however, that implicit in this reasoning is the notion that the representation of reality is bound to be approximate, which suggests that the object of inquiry, in the empirical sciences, is not completely graspable after all, but only approximately so.

This was just a first attempt to reflect philosophically on these complex issues, and Reichenbach will return to some of them soon afterwards, later abandoning the idea of convergence “with certainty” in favour of convergence “with probability”—but that is another story.

## Coordination refined

Reichenbach’s initial considerations on coordination are further developed in his Habilitation thesis, which extensively focuses on the idea of cognition *as* coordination (1920, ch. 4). The leitmotiv behind coordination is quite similar to that of his doctoral thesis, but now more clearly formulated in terms of mapping: both mathematical and physical types of coordination involve the coordination between two sets, except that only the mathematical ones are fully defined and can be coordinated without any other assertion. Physical coordination, instead, primarily involves the relation of concepts to their corresponding intuitional contents, which, as we have seen, is particularly important when it comes to individuating, measuring, and defining physical magnitudes. But now the idea of coordination takes a more explicit holistic turn (Padovani, 2011). As Reichenbach argues (1920, p. 37; emphasis in the original):

The physical relation can be conceived as a coordination: physical things are coordinated to equations. Not only the totality of real things is coordinated to the total system of equations, but *individual* things are coordinated to *individual* equations. The real must always be regarded as given by some perception. […] If we speak of Boyle’s gas law, we coordinate the formula *pV* = *RT* to certain perceptions, some of which we call direct perceptions of gases (such as the feeling of air on the skin) and some of which we call indirect perceptions (such as the position of the pointer of a manometer). The fact that our sense organs mediate between concepts and reality is inherent in human nature and cannot be refuted by any metaphysical doctrine.

The problematic but fundamental feature of physical coordination is that only one of the two sides of the coordination is defined (as it is expressed in formal / mathematical terms) while the undefined side, the one dealing with the “cognition of reality” is “completely undefined. It is not delimited, it contains no direction, and it does not even give a clue as to what constitutes an individual element of the set.” In other terms, the real is per se not attainable and no specifications can be provided that would successfully secure a physical coordination once and for all (pp. 38–40). One could be tempted to appeal to “perception” in order to determine the “real”, but Reichenbach clarifies that this is not possible, and the reason is simple: perceptions are complex and in order to coordinate them, we should first be in a position to only consider their significant components while removing the irrelevant ones. However, this already requires a great deal of theory and reasoning, which means that perception *alone* really cannot provide any direct access to, or whatsoever definition of, what is real. So, how is physical coordination supposed to function as an effective mapping strategy if one of its sides is not determined? For this purpose, Reichenbach introduces a new salient feature: that of “mutuality of coordination”. As he tries to explain (1920, p. 42; emphasis in the original):

Although there is a coordination to undefined elements, it is restricted, not arbitrary. This restriction is called ‘the determination of knowledge by experience’. We notice the strange fact that it is the defined side that determines the individual things of the undefined side, and that, vice versa, it is the undefined side that prescribes the order of the defined side. *The existence of reality is expressed in this mutuality of coordination*.

The idea, on which Reichenbach does not elaborate much at all, unfortunately, is that the undefined side providing the empirical content shapes our physical coordination as much as our physical coordination is shaped by, and in turn conforms to, our empirical reality.^26^ This model requires constant refinement, i.e., an *iterative* procedure, systematic empirical confirmation in deploying physical coordination, and in addition to this, a criterion that defines when a coordination is successful. To be sure, this criterion is external to the process of coordination and involves a concept we have already seen when discussing the measurement of amplification, that of “univocality” or “uniqueness”. In Reichenbach’s words: “*Uniqueness* [*Eindeutigkeit*] of a cognitive coordination means that a physical variable of state is represented by the *same* value resulting from different *empirical data*”. Perceptions cannot determine what is real, but they can help us establish whether a coordination is univocal (*eindeutig*), namely requiring that, “with respect to an individual process, the value of the constants be completely determined by all factors, including, in a given case, the coordinates. This requirement must be satisfied; otherwise, the numerical value of the variable of state cannot be calculated by a chain of reasoning and experience” (1920, pp. 44–46).

As physical statements are expressed in mathematical form, and they do not per se include an assertion concerning their empirical validity, we need to presuppose some coordinating principles, which operate as “prescriptions for the conceptual side of coordination” allowing us to meaningfully connect those equations with pieces of reality. These principles must thus function as “rules of the application”, as Reichenbach puts it, of abstract structures to real ones.^27^ In the Kantian language that Reichenbach is still using, this implies that those principles are “constitutive” of empirical statements, ultimately determining their validity. Without these principles, which are theory-specific, it would not be possible to even deem an empirical statement meaningful at all.^28^ Despite displaying a foundational nature, principles of the physical coordination are not regarded as “immutable”: they are necessary to carry out the scientific enterprise but not forever and only so long as they enable us to do so. This means that these principles can be revised should inconsistencies arise, which would lead us to modify our theory and ultimately to scientific change. It goes without saying that the idea of “univocality” is essentially a criterion for validity. Yet, in Reichenbach’s (1920), the idea of empirical validity is not presented as pre-defined as it depends on the set of principles that are constitutive in each specific stage. Since the possibility of scientific change can never be dismissed, we have to assume that we can always substitute old foundational principles with new ones, which should possibly incorporate them.^29^ Consistently with this reasoning, Reichenbach goes so far as to admit that even the criterion of uniqueness, like any other principle, could be given up in the future if scientific progress required us to do so.

Although this model may look like involving a circular procedure, it actually avoids the circularity problem. Reichenbach also does not propose what we could define as a coherentist account.^30^ These coordinating principles are foundational and theory-specific, even though they operate in a contextual manner. Changing them entails a change in our concept of knowledge and results in a new object of knowledge, which now “*presupposes different logical conditions*” (1920, p. 104). Even more: knowledge can only be achieved gradually: “*even our concepts of the very object of knowledge, that is, of reality and the possibility of its description, can only gradually become more precise*” (1920 p. 88, both emphases in the original).

However, how is it possible to truly conceive of a new scientific stage, i.e., revise and accordingly substitute our foundational principles from within? For Reichenbach, when a set of principles, considered in their totality, cannot solve a contradiction—or enable a path for disentangling inconsistencies without severely modifying the set (or the very nature) of those principles—typically a new principle is introduced that makes the contradiction “disappear”. This can only happen if the old principle is an approximation (*Näherung*) for some simple cases: “Since all experiences are merely approximate laws (*Nährungsgesetze*), they may be established by means of the old principles” and we cannot “exclude the possibility that the totality of experiences inductively confirms a more general principle”. And he concludes: “*It is logically admissible and technically possible to discover inductively new coordinating principles that represent a successive approximation of the principles used until now*” (1920, pp. 68–69; emphasis in the original).^31^

The next question would now be: how can we confirm our scientific inferences and the principles they rely on? In a paper written in 1923 (but only published in 1932), Reichenbach broadly addresses these issues and articulates how theory testing can show whether even a fundamental principle such as causality can be confirmed or disconfirmed in the future (as it will turn out). He analyses various meanings of “causality” and focuses on the most important one at the core of scientific reasoning, i.e., what he calls “the inductive principle of causality”. This principle asserts that natural events are connected in a way that allows us to predict unknown event from known ones.^32^ As he contends, “the predictability of *unobserved* events by means of *observed* ones is the central problem of causality” and it crucially depends on the existence of a functional relationship, basically an extrapolation, linking the unobserved state (to be experienced in the future) with the observed one (1923/1932, p. 346). Reichenbach’s formulation of this principle is once again reflecting his peculiar methodology informed by experimental practice and it is admittedly “guided by the procedure that the physicist uses in order to establish an individual law”: a function is initially formulated as a hypothesis, and it is subsequently tested by experiments. Depending on whether measurements and tests provide a “quantitative confirmation”, the “hypothesis will be regarded as true or false” (1923/1932, p. 346). We will not follow Reichenbach’s quite tangled reasoning in this paper,^33^ but the take-home message here is that even a pivotal principle such as causality could be dismissed, as it is “not impossible that physics will someday be confronted by phenomena that compel it to abandon causality” (1923/32, p. 370).^34^

## Coordination and validity

After examining Reichenbach’s approach to measurement, coordination, and testing we can retrospectively reflect on how current discussions of validity in human sciences, particularly psychology, may draw analogies to his ideas. While there does not seem to be complete agreement on the meaning of “construct validity” (Stone, 2019; Feest, 2020), several aspects that are considered in the literature resonate particularly well with Reichenbach’s interpretation.^35^ My aim here is not to articulate these analogies precisely, but simply to hint at the conceptual proximity between coordination and construct validity.

The topics covered in the previous sections involve several types of research and span across various fields. Nonetheless, Reichenbach’s attitude with respect to coordination (even in its rudimentary version) points to the same upshot, namely, that the application of mathematical structures to reality needs well more than a good mathematical model that appears to “fit” the phenomena: it needs a great deal of prior theoretical thinking and interpretive judgement to implement any coordination. Reichenbach’s emphasis on the individuation of quantities through a progressive refinement process enabling us to define (and thus measure) physical magnitudes is a crucial part of his account. As we have seen in his treatment of the definition of amplification, as well as in his refined framework of coordination in 1920, he was reflecting on the type of work required to establish—at least provisionally—core concepts, or what we would now refer to as “theoretical constructs”.

It is noteworthy that some of these ideas have to date been explicitly evoked in the framework of psychology, which is one of the early landscapes in which Reichenbach was operating. For instance, in their overview of the formalization of psychological theory, Eronen and Romeijn (2020) have recently referred to Reichenbach’s interpretation of coordination and its role in linking abstract concepts with their empirical correlate as providing an inspiring solution to address some open issues, especially in relation to the scarce examination of concept definitions in psychology.^36^ Interestingly, they invoke the same type of “coordinative labor” that Reichenbach was elaborating upon especially in his early work. As they explain (2020, pp. 793–794):

concepts in psychology are often given operational definitions, which do not allow for much epistemic iteration. However, even more common in contemporary psychology is that concepts are not explicitly defined at all. […] In other words, the sort of co-ordination or epistemic iteration we have emphasized in our discussion of the mathematization of science has hardly taken place in psychology. […] There have been extensive discussions throughout the decades on construct validation and psychological measurement, but they have not resulted in sustained efforts to coordinate theoretical concepts with the empirical world through an iterative process. […] Looking at the introduction and deployment of statistical methods and representations into psychology, we can safely say that they did not take up a role as representations of reality. The Reichenbachian analysis of how hypotheses apply to the empirical domain of psychology indeed seems more apt: a lot of ‘co-ordinative labor’ has to be carried out to match the hypotheses to empirical patterns.

Hence, the need to specify coordinative definitions in order to fully capture the relevant empirical structure. Without precise definitions, these concepts could not be properly employed in mathematical structures. To be sure, the type of quantities and magnitudes Reichenbach discussed were not as potentially complex or problematic as those addressed in contemporary literature on construct validity—such as happiness, well-being, intelligence, or other psychological attributes—which clearly pose challenges for quantification. Yet, the type of iterative procedure suggested is quite similar: knowledge through coordination can only be achieved gradually, by progressively shaping the unformed material from perception into better defined quantities by means of coordination (and the “mutuality” that comes with it). In a recent paper, Feest (2020) emphasizes that also “construct validity is a matter of degrees” (sec. 5) and argues that “psychological constructs often start their lives as pre-theoretical or folk-psychological concepts, which can be […] improved as part of an iterative process, whereby the initial construct informs a test, which in turn can contribute to the further development of the construct, relative to which the construct validity of the test can be investigated, in turn enabling additional development/refinement of the constructs” (p. 4). In a similar vein, Stone (2019, p. 1258) maintains that “both validity and legitimacy are evolving properties, such that a construct or measure may become more or less legitimate / valid over time as new evidence is gathered.”

In addition, even when a coordination is successful, it does not mean that it will always be so, and that it may just need to be slightly modified or adapted in the future if needed. Reichenback clearly contemplates the possibility that a system of coordinating principles and hypotheses could be heavily revised (if not rejected), if it no longer described the world in a meaningful way. As we have seen, certain inconsistencies could indeed arise, requiring a significant change in the theoretical framework. This, in turn, would then lead to a new set of coordinating principles, thereby defining a new concept of the object of knowledge. From this perspective, it is evident that Reichenbach’s early account of cognition as coordination leaves little room for a realist position.^37^ Similarly, even though measurement validity and scientific realism have often been associated in validity theory, also “(construct) validity” appears to be primarily an epistemic concept (Hood, 2009).

Another aspect emphasized in the literature on construct validity, particularly pertinent to our discussion, is the idea, which aligns with Reichenbach’s, that the choice of coordination can (and actually does) affect not only the theory but also theory testing. Kellen et al. (2021) have recently reinforced the importance of the choice of coordination in theory testing, because “the testability of a theory is codetermined by the structure of the latent variables that it postulates” and “the coordination functions that are imposed”. Furthermore, using a case-study based on the face-inversion effect, they have shown “how the problem of coordination can severely affect the conclusions that can be drawn from observed pattern of data” (2021, p. 774).^38^

Last, but not least, Zhao (2023) discusses a “thin conception of validity” that is well suited to capture the overall spirit of Reichenbach’s early practice-oriented, contextual account. As Zhao puts it, according to this conception, we “are no longer justified in inferring anything straightforward about the world or about the test from a claim of validity alone because claims of validity are always relativized to specific interpretations and use contexts” (p. 238), as we have seen was indeed the case of Reichenbach’s theory-specific conception.

In conclusion, the rich context in which Reichenbach initially developed the notion of coordination clearly shaped his approach, whose originality lies less in the attempted solutions themselves—which were not always successful—and more in the innovative way he integrated multiple strands of research and experimental practice. This is one of the reasons why the concept of coordination can be fruitfully applied across different disciplines, particularly in psychology, where it appears to fit naturally.^39^  

## Footnotes

1 See, for instance, van Fraassen (2008), Tal (2020), and literature therein.

2 See, for instance, Friedman (2001, p. 78) and in part also Ryckman (1991). In recent years, this interpretation has been extensively reconsidered as new studies have focused on Reichenbach’s early work also by exploring additional archival material (Ryckman, 2005; Padovani, 2011, 2017).

3 The institutionalization of psychology in Germany has a fascinating history, which ultimately has some relevance to Reichenbach’s academic curriculum. However, the lack of separation of psychology from philosophy was in part due to contingent events. In the early nineteenth century, in fact, the reformation of the German educational and university systems prompted several important changes in laws and regulations that resulted in the institution of a new qualifying examination in psychology for aspiring teachers at *Gymnasia*, on the one hand, and in the addition of philosophical propaedeutics (crucially including logic and psychology) to the *Gymnasium* curriculum, on the other; hence, the need for psychology courses. Besides, also physicians were required to take an examination in psychology, although this requirement did not immediately imply the creation of university chairs in that field. As Gundlach (2012, p. 264 ff.) observed, “what came into existence thanks to these reforms was not yet an independent discipline of psychology, but something we might call an auxiliary discipline”, whose aim was mainly to train “future teachers and physicians”, and not to “train psychologist at all […] since there were no chairs of psychology [… and] no social group responsible exclusively for psychology. Those responsible for teaching psychology had other, more important responsibilities, namely for philosophy and its main branches”. Thus, philosophers were in charge of teaching psychology on top of more traditional topics such as logic, history of philosophy, ethics and metaphysics.

4 Among these fields—noticeably, for our purposes here—physiology. See below, Sect. 2.2, as well as Gundlach (2012, pp. 268–273).

5 A byproduct of this development, depending on whether philosophy professors “had a psychological laboratory and knew how to do experimental research” or not, was, as Gundlach put it, an “informal division of labor” among philosophy chairs with one “specializing in metaphysics and history of philosophy, the other in psychology, pedagogics and philosophy of science, then usually called logic” (2012, p. 273).

6 This is not to imply that there was one “experimental method” that was uniformly applied or to suggest a clear distinction, in today’s terms, of what would be defined as “scientific” as opposed to “humanistic”. To the contrary, there were quite distinct approaches, ranging—just to mention a few—from Wilhelm Wundt’s systematic and experimental method rooted in introspection, to Gustav Fechner’s more quantitative and experimental psychophysics, to Hermann von Helmholtz’s empirical approach to physiology aimed at showing that mental processes have measurable physical correlates, to Franz Brentano’s more descriptive psychology, rejecting Wundt’s experimental introspection while focusing on mental acts and their intentionality. See Gundlach (2012). I am grateful to one of the reviewers for pressing me on this point.

7 Stumpf is the founder of the Berlin Institute of Psychology. His leadership allowed him to assemble a cohort of future leading figures of the new current of Gestalt psychology, like Wolfgang Köhler, Kurt Koffka, Max Wertheimer, and Kurt Lewin. Interestingly, Köhler and Lewin both worked in close connection with the Berlin Group of logical empiricists, and when Reichenbach was forced to leave Germany as a consequence of the rise of the Nationalist Socialist Party in 1933, Köhler (together with Nikolai Hartmann) took up the (nominal) supervision of one of Reichenbach’s most famous students, and later prominent philosopher of science, Carl Hempel.

8 As is well known, Stumpf was a student of Brentano, and heavily influenced by his teachings—as were, directly or indirectly, several later members of the Vienna Circle and the Berlin Group. See Smith (1994). Brentano was extremely instrumental in establishing psychology as a scientific discipline, and in emphasizing the primacy of direct experience and descriptive methods in research, all of which became central to Stumpf’s experimental approach, especially in his study of perception and sound. To be sure, Reichenbach did not show particular interest in Brentano’s work in relation to Stumpf’s. For the most part, Reichenbach critically discusses Stumpf’s subjective view of probability in his (1916), rejecting his interpretation of probability statements as basically expressing degrees of beliefs, and not depicting actual matters of fact in the world. See below, Sect. 2.2.

9 While elaborating on issues related to the mathematical representations of reality, and thus on coordination, Reichenbach does not engage with any other broader discussion, for instance, involving mental acts and mental phenomena in general, or even touching on the foundations of psychology. As we will see, his work on acoustic devices has more to do with amplifying valves and the measurement of amplification rather than with the nature and psychology of perception.

10 Reichenbach had to complete empirical research, which required interviewing and distributing a questionnaire to eleven-year-old children. This research, entitled “Psychologische Untersuchungen an Volkshochschulkindern”, was carried out during the winter semester 1912-13, and aimed to analyze the children’s attitudes, judgements, and psychological reactions to illicit actions. This hands-on approach provided Reichenbach with his (most likely) first practical experience in research methodology, albeit at a rudimentary level. See Padovani (2022, Sect. 3).

11 As Lang & Corstjens (2022, pp. 111–112) explain, “psychological laboratories typically had a set of devices to record reaction times, movements, or perceptions. One characteristic of early experimental psychology that resulted from its development of psychophysiology and psychology was a keen interest in the scientific measurement of psychological states”. To be sure, in the mid of 1910s, Müller was best known for his works on the foundation of psychophysics and of memory, even though Reichenbach only attended his course on logic while in Göttingen.

12 At the same time, he was able to further pursue his academic interests in physics. In fact, as is well known, Reichenbach was among the first five students who attended Einstein’s lectures on special and general relativity, and on statistical mechanics in Berlin, between 1917 and 1920 (Padovani 2011, fn. 4).

13 Among others, Lipmann also developed the first selection tests for aviators, typesetters, and other industrial figures and was also one of the first psychologists to employ statistics in this work. Incidentally, the term “Psychotechnik” was apparently coined by Stern, and nicely “captured the idea that psychology could be utilized as a form of technology” (Lang & Corstjens, 2022). Stern is also renowned for inventing the IQ formula, and beyond diagnostics, his work on “personalistic psychology” (*Personalismus*) was pivotal in establishing “psychology of personality” as a discipline (Gundlach, 2012, pp. 275–278).

14 Their joint work resulted in a document, entitled “Entwurf zu einer Eignungsprüfung für Funkentelegraphisten”, signed by all three and completed in 1917. See Padovani (2013, p. 101).

15 Alan Richardson reports that “at one point in the project (1 August 1917), Lipmann felt called upon to admonish Reichenbach to keep in mind the practical import of the their work for the military, writing in a letter’It seems to me that in our many discussions the chief labour of our experiments has somewhat fallen out of sight: we must again remember that our goal is not a scientific investigation but rather a practical task’.” Richardson (2021, p. 12,629).

16 See Padovani (2013, p. 101). Reichenbach used some of these technical reflections for his volume on radio, in which, among other things, he discussed sound transmission and reception illustrating devices from the company Huth (e.g., Reichenbach, 1924, p. 57).

17 These materials are available in the Hans Reichenbach Papers, 1884-1972, ASP.1973.01, at the Archives of Scientific Philosophy, Archives & Special Collections, University of Pittsburgh Library System. In what follows, references to archival materials will indicate the abbreviation for the Collection (HR), followed by the number of the box, folder, and item, respectively. Unless otherwise indicated, all translations from this Collection are mine.

18 Reichenbach’s reasoning is the following: “The primary power at the input transformer depends on current, voltage, and phase. If the phase angle is increased, the same power can be achieved with a higher voltage. It is quite possible that in this case, the amplification factor will be different. The amplifier is a relay and can output arbitrarily high power. The amount of output power is entirely independent of the primary power itself. What matters is how the primary power occurs. If the voltage is higher with the same primary power, the output power is probably higher, as the tube responds to voltage at the grid” (HR 018-14-01, p. 2).

19 As is well known, Kant’s original deterministic framework would not naturally allow for probability in the sense that probability is not part of the fundamental way in which the mind structures experience. In Reichenbach’s interpretation, however, the very possibility of using mathematics to formulate laws in our representation of reality requires the type of certainty that the traditional set of Kantian principles cannot fully support as we can only rely on a finite number of repetitions of cases for which we need to be able to deal with approximations. The aim of Reichenbach’s doctoral thesis is in fact to show the existence of a “principle of probability”, which is needed to complement the more traditional “principle of causality” (or “principle of lawful connection” as he calls it). The principle of probability, which he refers to as “the principle of lawful distribution” and that he considers to be “synthetic a priori”, should guarantee the existence of a convergence limit of the empirical distribution of values. See below, footnote 25.

20 Similarly, the quantification problem in psychophysics and, more generally, discussions on Fechner’s psychophysical law provided great impulse to a renewed reflection on measurement, especially in relation to the possibility of more accurate or reliable measurements of more complex magnitudes (Heidelberger, 2004). Hermann von Helmholtz’s monumental work in relation to the quantification problem is also emblematic in this sense (Biagioli, 2023). Notice that Reichenbach considered Helmholtz “the last of the universal physicists, the free-ranging spirits (…), who was able to combine experimental and theoretical physics” (Reichenbach, 1929, p. 247). Incidentally, when Moritz Schlick and Paul Hertz published an edited volume containing Helmholtz’s most influential epistemological writings (Helmholtz, 1921), Reichenbach wrote a review in Lipmann’s journal, the *Zeitschrift für angewandte Psychologie*, of all venues (Reichenbach 1921).

21 Among Reichenbach’s dissertation notes, there is a drawing of this device (HR 025-04-01) which he ultimately did not include in the dissertation. See also Eberhardt and Glymour’s 2008 introduction to Reichenbach’s doctoral dissertation translation (Reichenbach 1916/2008, p. 7), where they provide an illustration of it.

22 His analyses are reminiscent of and heavily rely on arguments from Poincaré’s method of the arbitrary function. See Eberhardt (2011).

23 Reichenbach’s aim is indeed to show that the equal size of the stripes is not the main condition in order to obtain the expected probability distribution.

24 The concept of “functional connection” or “functional relationship”, which is behind Reichenbach’s initially limited interpretation of causality, is derived from Cassirer’s most influential work *Substance and Function* (1910). Reichenbach does not elaborate much on this idea in this thesis, but it is clear that it associates it with a broader notion of “causal connection”, which is similar to the one discussed by Fechner, and later developed by Ernst Mach, at least in two respects: in relation to measurement, and in relation to the law of nature (but not in relation to the mind–body problem). On functional and causal relations in Fechner and Mach, see Heidelberger (2010). Reichenbach will carry out a more explicit discussion of causality in terms of “functional relationship” in his (1923/1932, Sect. 1). See also below, Sect. 3. It is worth mentioning that the interpretation of the nature of causality as a functional relationship was also part of debates that progressively lead to a distancing between Reichenbach and Schlick, starting in the mid-1920s. See Padovani (2010).

25 As Reichenbach puts it, “if there were a fully exact measurement of real things, then one should be able to claim that the value obtained once can be found again at any time at any place. Since we cannot claim that the value remains constant, we have no choice but to assume that if it is not constant then there exists some law for its distribution in time and space. This is the synthetic a priori judgment which we must make. It should be added that the same judgment must hold for the combination of several processes. As before, we cannot claim that the calculated value remains constant, and we have to replace it with its lawful distribution in space and time. Hence, we conclude that the principle of lawful connection of all events, which causality brings about, is insufficient for the mathematical representation of reality. A further principle has to be added, which connects the events—one could say—orthogonally; this is the principle of lawful distribution” (Reichenbach, 1916/2008, p. 127). For an analysis of how Reichenbach reaches this result and how he justifies this principle from a “Kantian” perspective, see again Eberhardt (2011).

26 Eberhardt (2022) provides an excellent illustration of Reichenbach’s account of coordination (including this feature) in showing how to identify and construct causal variables in recent research. See also Padovani (2017).

27 More specifically, coordinating principles must differ from physical laws in that “they do not connect certain variables of state with others but contain general rules according to which connections [i.e., what is expressed by a physical law] take place” (Reichenbach, 1920, p. 54).

28 Reichenbach provides a non-exhaustive list of coordinating principles and discusses why they should be deemed “constitutive” or foundational, albeit only relative to a specific scientific stage. See Padovani (2021) for a thorough discussion of Reichenbach’s interpretation of coordination.

29 Reichenbach extensively discusses the example of Euclidean metric, which, in “traditional physics” played the role of a coordinating principle because it constituted part of the concept of a physical thing, allowing for the combination of space points to create extended structures, independent of their physical properties. In general relativity, instead, the metric no longer performs that role, but that of a connecting principle. Cf. Reichenbach (1920, chap. VIII).

30 One such account of measurement, coherentist and model-based, is articulated in Tal (2019).

31 Reichenbach calls this inductive procedure a “method of continuous expansion” (*Verfahren der stetigen Erweiterung*), which has been quite loosely translated as “method of successive approximation”.

32 Fundamentally, this is what a scientific law amounts to for Reichenbach, namely: “a law is not a *description of what is observed*, but *of what is observable*” (1923/32, p. 357).

33 See Glymour (1979) and Padovani (2015).

34 It is remarkable that this conclusion is stated as early as 1923, before the advent of quantum mechanics would eventually confirm it.

35 These aspects go beyond Borsboom et al.’s (2009) characterization of the broad connection between the philosophical background of construct validity theory and the overall logical empiricist account, as they put it in (2009, p. 136): “Construct validity theory, as Cronbach and Meehl (1955) introduced it, holds that a test score interpretation in terms of nomological network (= a set of laws relating theoretical terms in that network to each other and to observational terms) is valid to the degree that the network itself is supported by the evidence. This idea leaned heavily on the philosophical framework provided by logical positivism, which used the same construction as a general account of the relation between theoretical terms and observations in scientific theory.”

36 As they acknowledge, “the Reichenbachian analysis highlights the constructive role that mathematics plays in identifying empirical patterns and salient concepts that can feature in the definitions, and make them fully precise in mathematical terms” (2020, p. 790).

37 And it is not surprising that his early approach has been appealing to some anti-realists (van Fraassen, 2008; Padovani, 2017).

38 One of the motivations for Kellen et al.’s paper is to reject operationalism in measurement. Because operationalism defines measurement as “any specified operation that yields a number”, it ignores the problem of coordination, and it “effectively enforces a complete disconnect with natural reality: We are no longer concerned with the ability of Y capturing some unobservable X—quantity Y has become its own measure. It also follows that any concept of validity has to be discarded because we can no longer evaluate the consistency of measurements coming from different instruments: They measure different things simply because they involve different operations—a vicious circularity” (2021, p. 772).

39 An excellent recent example is Luchetti (2021).
